# Effectiveness of BNT162b2 and CoronaVac vaccinations against mortality and severe complications after SARS-CoV-2 Omicron BA.2 infection: a case–control study

**DOI:** 10.1080/22221751.2022.2114854

**Published:** 2022-10-09

**Authors:** Vincent Ka Chun Yan, Eric Yuk Fai Wan, Xuxiao Ye, Anna Hoi Ying Mok, Francisco Tsz Tsun Lai, Celine Sze Ling Chui, Xue Li, Carlos King Ho Wong, Philip Hei Li, Tiantian Ma, Simon Qin, Vincent Kai Chung Wong, Tat Chi Tsang, Sik Hon Tsui, William Chun Ming Chui, Benjamin John Cowling, Gabriel Matthew Leung, Chak Sing Lau, Ian Chi Kei Wong, Esther Wai Yin Chan

**Affiliations:** a Centre for Safe Medication Practice and Research, Department of Pharmacology and Pharmacy, Li Ka Shing Faculty of Medicine, The University of Hong Kong, Hong Kong, People’s Republic of China; b Laboratory of Data Discovery for Health (D^2^4H), Hong Kong Science and Technology Park, Hong Kong, People’s Republic of China; c Department of Family Medicine and Primary Care, School of Clinical Medicine, Li Ka Shing Faculty of Medicine, The University of Hong Kong, Hong Kong, People’s Republic of China; d School of Nursing, Li Ka Shing Faculty of Medicine, The University of Hong Kong, Hong Kong, People’s Republic of China; e School of Public Health, Li Ka Shing Faculty of Medicine, The University of Hong Kong, Hong Kong, People’s Republic of China; f Department of Medicine, School of Clinical Medicine, Li Ka Shing Faculty of Medicine, The University of Hong Kong, Hong Kong, People’s Republic of China; g Department of Pharmacy, Queen Mary Hospital, Hong Kong, People’s Republic of China; h Department of Accident and Emergency, Queen Mary Hospital, Hong Kong, People’s Republic of China; i World Health Organization Collaborating Centre for Infectious Disease Epidemiology and Control, School of Public Health, Li Ka Shing Faculty of Medicine, The University of Hong Kong, Hong Kong, People’s Republic of China; j Research Department of Practice and Policy, School of Pharmacy, University College London, London, UK; k Aston Pharmacy School, Aston University, Birmingham, UK; l Department of Pharmacy, The University of Hong Kong-Shenzhen Hospital, Shenzhen, People’s Republic of China; m The University of Hong Kong Shenzhen Institute of Research and Innovation, Shenzhen, People’s Republic of China

**Keywords:** COVID-19, Vaccine effectiveness, Omicron BA.2, CoronaVac, BNT162b2

## Abstract

Data regarding protection against mortality and severe complications after Omicron BA.2 infection with CoronaVac and BNT162b2 vaccines remains limited. We conducted a case–control study to evaluate the risk of severe complications and mortality following 1–3 doses of CoronaVac and BNT162b2 using electronic health records database. Cases were adults with their first COVID-19-related mortality or severe complications between 1 January and 31 March 2022, matched with up-to 10 controls by age, sex, index date, and Charlson Comorbidity Index. Vaccine effectiveness against COVID-19-related mortality and severe complications by type and number of doses was estimated using conditional logistic regression adjusted for comorbidities and medications. Vaccine effectiveness (95% CI) against COVID-19-related mortality after two doses of BNT162b2 and CoronaVac were 90.7% (88.6–92.3) and 74.8% (72.5–76.9) in those aged ≥65, 87.6% (81.4–91.8) and 80.7% (72.8–86.3) in those aged 50–64, 86.6% (71.0–93.8) and 82.7% (56.5–93.1) in those aged 18–50. Vaccine effectiveness against severe complications after two doses of BNT162b2 and CoronaVac were 82.1% (74.6–87.3) and 58.9% (50.3–66.1) in those aged ≥65, 83.0% (69.6–90.5) and 67.1% (47.1–79.6) in those aged 50–64, 78.3% (60.8–88.0) and 77.8% (49.6–90.2) in those aged 18–50. Further risk reduction with the third dose was observed especially in those aged ≥65 years, with vaccine effectiveness of 98.0% (96.5–98.9) for BNT162b2 and 95.5% (93.7–96.8) for CoronaVac against mortality, 90.8% (83.4–94.9) and 88.0% (80.8–92.5) against severe complications. Both CoronaVac and BNT162b2 vaccination were effective against COVID-19-related mortality and severe complications amidst the Omicron BA.2 pandemic, and risks decreased further with the third dose.

## Introduction

The Omicron variant of severe acute respiratory syndrome coronavirus 2 (SARS-CoV-2) is considered more transmissible compared to other previously known variants of the virus, with an estimated effective reproduction number 3 times greater than that of the Delta variant [1]. The spread of the Omicron variant has resulted in among the greatest wave of the coronavirus disease 2019 (COVID-19) pandemic, even in countries with prior successful control of the outbreak such as China and New Zealand. After more than 6 months of almost zero new cases of COVID-19 and related deaths in 2021, Hong Kong faced a major outbreak with more than 50,000 new cases and close to 300 deaths recorded daily at its peak in early March 2022 [2], despite pre-existing stringent border control, implementation of social distancing, quarantine and contact tracing measures. With 7825 COVID-19-related deaths recorded by the end of March 2022 in a population of 7.4 million, Hong Kong observed the world’s highest COVID-19 fatality rate [3]. Globally, the cumulative number of confirmed cases reached 500 million, and is still on the rise despite high COVID-19 vaccination coverage [4]. Together with the notably high mortality rate recorded locally, this has led to debate regarding the real-world effectiveness of currently available vaccines against the Omicron variant.

The COVID-19 mass vaccination programme in Hong Kong commenced on 26 February 2021, with two vaccines, namely BNT162b2 from Fosun-BioNTech (Pfizer-BioNTech, mRNA vaccine) and CoronaVac from Sinovac Biotech (HK) Limited (inactivated vaccine). While the efficacy of BNT162b2 against severe disease and mortality with COVID-19 is well established based on previous large-scale randomised trials and observational studies [5–8], evidence regarding protection against the Omicron variant, specifically, remains limited in scope. Recent studies suggest that the effectiveness of BNT162b2 against Omicron was reduced, although effectiveness against severe disease and death remained relatively high in those who had received a booster dose [9–11]. More importantly, these studies mainly focused on the BA.1 lineage of the Omicron variant and non-Chinese population, whereas the BA.2 lineage is the dominant strain in the current wave of outbreak in Hong Kong, China [12] and worldwide [13].

Data on the inactivated vaccines’ effectiveness against Omicron remains scant. Several observational studies in Chile, Brazil and China showed effectiveness of two doses of inactivated vaccine ranging from 81.3 to 100% against severe COVID-19 before the Omicron outbreak [14–16]. Although studies have investigated the level of serum neutralising antibodies in CoronaVac recipients [17], no real-world studies with clinical outcomes in the Omicron pandemic have been published so far. Most importantly, inactivated COVID-19 vaccines contributed to almost half the total number of COVID-19 vaccine doses administered globally [18], and therefore this study will address an important knowledge gap regarding vaccine effectiveness against Omicron BA.2.

The recent Omicron BA.2 dominant outbreak has resulted in an extraordinary high mortality rate in Hong Kong. Along with the suboptimal vaccination coverage locally, this represents a unique situation where sufficient real-world data was available to evaluate the vaccine effectiveness against severe disease caused by Omicron BA.2. Based on vaccination records from the Department of Health, only 63% of the Hong Kong population had received at least two doses of COVID-19 vaccine by the end of 2021. By comparing the risk of severe COVID-19 and COVID-19-related mortality among those vaccinated with one to three doses of BNT162b2 or CoronaVac, versus those who remained unvaccinated, this study aims to evaluate the real-world effectiveness against severe or fatal COVID-19, following vaccination of the two vaccines.

## Methods

### Study design and population

This case–control study included individuals aged ≥ 18 years. Cases were adults with their first COVID-19-related mortality or severe complications between 1 January and 31 March 2022, matched with up-to 10 controls by age, sex, index date and Charlson Comorbidity Index. This study was conducted using routine electronic health records from the clinical management system under the Hospital Authority (HA) of Hong Kong and vaccination records from the Department of Health (DH) of the Government of the Hong Kong Special Administrative Region. These two databases are linked based on the unique Hong Kong Identity Card Number or other personal identification numbers. The HA is a statutory administrative organisation in Hong Kong that manages all public inpatient services and most public outpatient services. The clinical management system, which includes demographics, diagnoses, prescriptions and laboratory tests, provides real-time data support and monitoring for routine clinical management across all clinics and hospitals in HA. The DH manages and retains the database for all vaccination records in Hong Kong. The two population-based databases have been used previously to conduct studies on the risk of adverse effects after COVID-19 vaccinations and other COVID-19 pharmacovigilance studies [19–27].

### Definitions of vaccine exposure

Two COVID-19 vaccines, BNT162b2 and CoronaVac, have been available under emergency use for individuals aged 16 years old or above in Hong Kong since 23 February 2021, subsequently extending to individuals aged 12 years or above since June and November 2021 for BNT162b2 and CoronaVac, respectively. COVID-19 booster shots were made available for priority groups on 11 November 2021, and the scheme was subsequently expanded to the general population from 1 January 2022 [28,29]. Details of the rollout schedule are listed in Supplementary Table 1. Individuals have a choice between BNT162b2 or CoronaVac for their first dose and are restricted to the same vaccine for their second dose. For the third dose, individuals have a choice of either vaccine. Hence, in this study, COVID-19 vaccination status was classified into eight mutually exclusive groups based on the number of vaccine doses and vaccine type administered. The eight groups were (i) 1-dose-only BNT162b2, (ii) 1-dose-only CoronaVac, (iii) 2-doses-only BNT162b2, (iv) 2-doses-only CoronaVac, (v) 3-doses (all BNT162b2), (vi) 3-doses (all CoronaVac), (vii) 3-doses (2-dose BNT162b2 followed by CoronaVac), and (viii) 3-doses (2-dose CoronaVac followed by BNT162b2).

### Definitions of outcomes

The outcomes investigated in this study were (i) COVID-19-related mortality, defined as all-cause mortality within 28 days after COVID-19 infection, (ii) COVID-19-related severe complications, defined as admission to intensive care unit (ICU) or use of ventilatory support within 14 days after COVID-19 infection. COVID-19 infection was defined as a positive polymerase chain reaction (PCR) test using throat swab, nasopharyngeal aspirate, or deep throat sputum specimens. PCR test results were recognised as the gold-standard diagnostic criteria for COVID-19 infection and were provided by the Public Health Laboratory Services Branch, Department of Health, Government of Hong Kong SAR and the Hospital Authority of Hong Kong. The Hong Kong government has implemented extensive PCR testing for SARS-CoV-2 in public hospitals and clinics for close contacts with confirmed cases and those who presented with COVID-like symptoms. From the start of the pandemic until mid-February 2022 all confirmed cases including asymptomatic cases were strictly isolated in either hospital or a community isolation facility, but after mid-February mild cases were permitted to isolate at home. The government has also set up territory-wide community testing centres to screen asymptomatic individuals and provide regular testing to various staff groups with a high risk of exposure, such as those working in nursing homes. Information regarding all-cause mortality was extracted from the Hong Kong Deaths Registry, which is an official governmental registry covering all registered deaths for Hong Kong residents. Use of ventilatory support, including intubation, mechanical ventilation and oxygen supplementation, was identified using ICD-9-CM procedure codes (39.65, 89.18, 93.90, 93.95, 93.96, 96.04, 96.7x).

### Definition of cases and controls

To evaluate the association between vaccination and the risk of outcomes after contracting predominantly Omicron BA.2 variant [12], the inclusion period for each outcome was from 1 January 2022–31 March 2022. For COVID-19-related mortality, cases were those who died within 28 days after COVID-19 infection, and controls were selected from all other patients who attended any HA health services and were not cases. For COVID-19-related severe complications, cases were those admitted to intensive care unit (ICU) or used ventilatory support within 14 days after COVID-19 infection, and controls were selected from all other patients who attended any HA health services and were not cases. Patients with a history of COVID-19 infection before 1 January 2022, or incomplete vaccination records were excluded. Up to ten controls were randomly matched with the cases according to sex, age (5-year band), date of attendance (within three calendar days) and Charlson Comorbidity Index (0,1–2,3–4, ≥5) [30].

### Statistical analysis

Conditional logistic regressions adjusted for chronic comorbidities, including cancer, chronic kidney disease, respiratory disease, diabetes mellitus, cardiovascular disease, dementia; as well as the use of chronic medications including renin-angiotensin-system agents, beta-blockers, calcium channel blockers, diuretics, nitrates, lipid-lowering agents, insulins, antidiabetic drugs, oral anticoagulants, antiplatelets, and immunosuppressants, were applied to evaluate the association between vaccination status and the risk of COVID-19-related mortality and severe complications. Adjusted odds ratio (OR) with 95% confidence interval (CI) were reported. Vaccine effectiveness was estimated by (1 - adjusted OR) x 100%. Age-specific estimates by age group (18–50, 51–64, ≥65 years) were reported.

Three sensitivity analyses were conducted. First, as previous studies suggested that the vaccine was safe and immunogenic in most patients 14 days after receiving the second dose [31,32], vaccine recipients were defined as individuals who received the vaccination at least 14 days before the index date. Second, only hospitalised patients were included and a test-negative case control study design was used, where those who had a positive PCR result within 48 h of admission were regarded as cases, and those who had a negative PCR result within 48 h of admission were regarded as controls. Third, use of antibacterial and antiviral agents within the past 7 days were adjusted in the regression model to account for the possibility of any concurrent infections which may have led to adverse short-term outcomes.

Sample size and power estimation are shown in Supplementary Figure 1. 5–78 matched sets (each set has 1 case and 10 controls) were required to achieve 80% power to detect the odds ratios from 0.05–0.5 (corresponding to vaccine effectiveness of 50% to 95%) at 0.05 significance level. All statistical tests were two-sided, and *P* values less than 0.05 were considered statistically significant. Statistical analysis was conducted using R version 4.0.3 (www.R-project.org). At least two investigators (VKCY, EYFW, XY) conducted the statistical analyses independently for quality assurance. STROBE (Strengthening the Reporting of Observational Studies in Epidemiology) statement checklists were followed to guide transparent reporting of the case–control study ^20^.

### Ethics approval

This study was approved by the Central Institutional Review Board of the Hospital Authority of Hong Kong (CIRB-2021-005-4) and the DH Ethics Committee (LM171/2021).

### Role of the funding source

The funder has no role in the study design, data collection, data analysis, data interpretation and writing of the report. The corresponding authors had full access to all the data in the study and took final responsibility for the decision to submit for publication.

## Results

**Figure 1.**
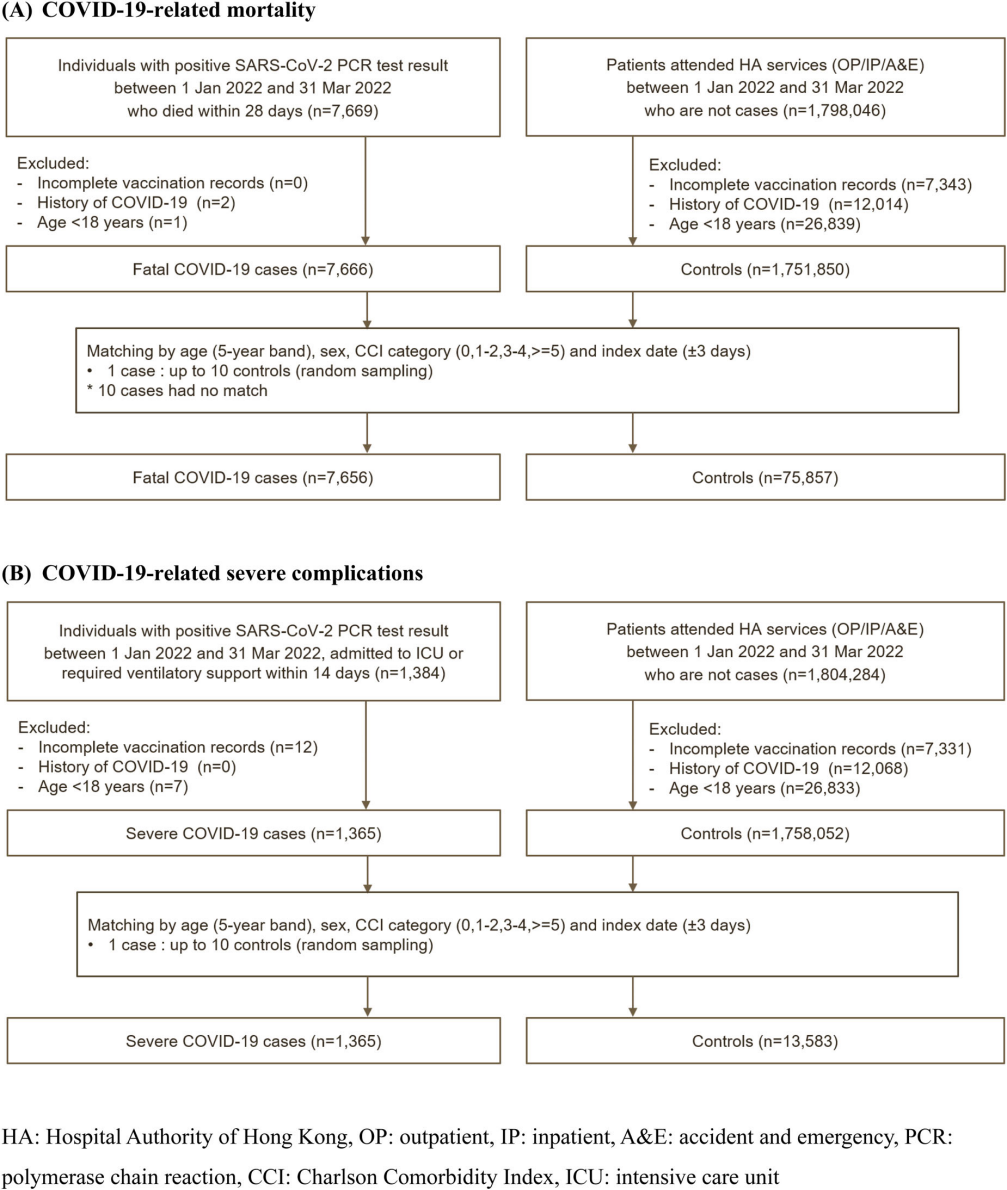
Selection of cases and controls.

A total of 7656 and 1365 cases of COVID-19-related mortality and severe complications were matched to 75,857 and 13,583 controls, respectively (Figure 1). Table 1 summarises the characteristics of the cases and controls.

**Table 1.** Baseline characteristics of cases and controls.

Outcomes	Mortality		Severe complications	
	Cases	Controls	Cases	Controls
Number of individuals	7656	75857	1365	13583
Age, years – mean (SD)	84.02 (11.31)	83.73 (11.10)	74.45 (15.49)	74.31 (15.37)
Sex, male – no. (%)	4530 (59.2)	45018 (59.3)	829 (60.7)	8264 (60.8)
Charlson Comorbidity Index – mean (SD)	1.78 (2.07)	1.65 (1.96)	1.38 (1.80)	1.30 (1.79)
Time since most recent dose – mean (SD)	51.67 (67.80)	56.07 (65.02)	61.91 (70.98)	71.02 (74.19)
**Pre-existing comorbidities – no. (%)**				
Cancer	762 (10.0)	7670 (10.1)	88 (6.4)	1144 (8.4)
Chronic Kidney Disease	972 (12.7)	9928 (13.1)	194 (14.2)	1260 (9.3)
Respiratory disease	846 (11.1)	6703 (8.8)	126 (9.2)	818 (6.0)
Diabetes	2207 (28.8)	28949 (38.2)	394 (28.9)	4813 (35.4)
Cardiovascular disease	4915 (64.2)	55240 (72.8)	762 (55.8)	8588 (63.2)
Dementia	668 (8.7)	2443 (3.2)	57 (4.2)	267 (2.0)
**Medication use within 90 days – no. (%)**				
Renin-angiotensin-system agents	2609 (34.1)	32152 (42.4)	495 (36.3)	5227 (38.5)
Beta blockers	2041 (26.7)	19443 (25.6)	388 (28.4)	3274 (24.1)
Calcium channel blockers	3630 (47.4)	44381 (58.5)	619 (45.3)	7014 (51.6)
Diuretics	2145 (28.0)	10735 (14.2)	332 (24.3)	1489 (11.0)
Nitrates	965 (12.6)	6928 (9.1)	153 (11.2)	964 (7.1)
Lipid lowering agents	3292 (43.0)	43678 (57.6)	601 (44.0)	7316 (53.9)
Insulins	999 (13.0)	4444 (5.9)	151 (11.1)	728 (5.4)
Antidiabetic drugs	1832 (23.9)	24271 (32.0)	362 (26.5)	4289 (31.6)
Oral anticoagulants	678 (8.9)	4736 (6.2)	105 (7.7)	658 (4.8)
Antiplatelets	3046 (39.8)	25389 (33.5)	450 (33.0)	3596 (26.5)
Immunosuppressants	95 (1.2)	330 (0.4)	33 (2.4)	106 (0.8)
Antibacterial agents (within 7 days)	1647 (21.5)	978 (1.3)	145 (10.6)	135 (1.0)
Antiviral agents (within 7 days)	392 (5.1)	1548 (2.0)	59 (4.3)	315 (2.3)

SD: standard deviation, no.: number of individuals.

**Figure 2.**
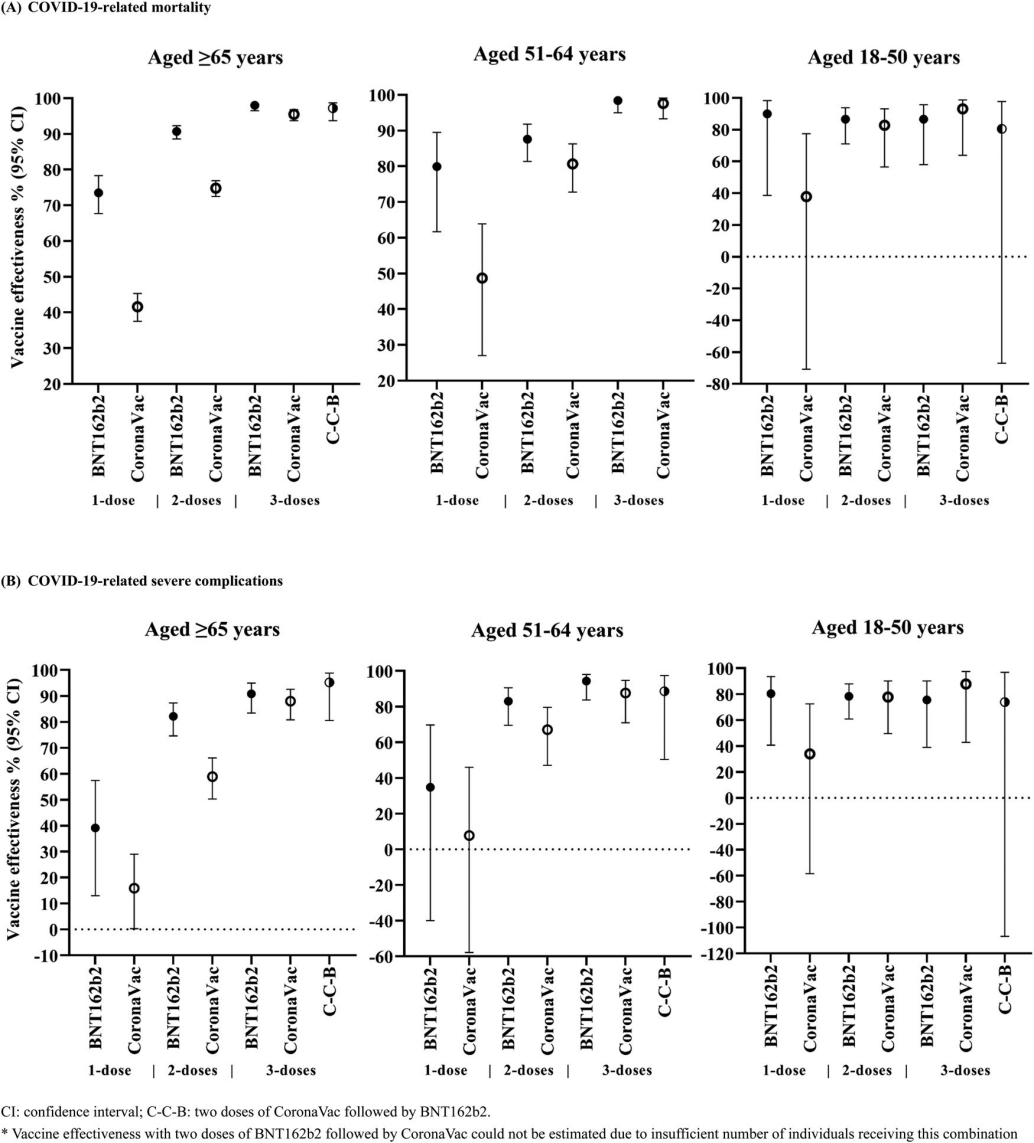
Vaccine effectiveness against COVID-19-related mortality and severe complications among individuals with different vaccination status by age group.

Tables 2 and 3 and Figure 2 show the adjusted odds ratios (ORs) and vaccine effectiveness (VE) for COVID-19-related mortality and severe complications. Both BNT162b2 and CoronaVac vaccination were associated with a significantly lower risk of COVID-19-related mortality and severe complications compared to unvaccinated patients, and the risk substantially decreased with an increasing number of doses.

**Table 2.** Vaccine effectiveness against COVID-19-related mortality among individuals with different vaccination status by age group.

Vaccination status	Case	Control	Crude OR (95% CI)	Adjusted OR (95% CI)	VE % (95% CI)
***Aged 65 years and above***					
Unvaccinated	4860	26990	(Ref)	(Ref)	(Ref)
*1 dose only*					
BNT162b2	111	2218	0.244 (0.201–0.296)	0.265 (0.217–0.323)	73.5 (67.7–78.3)
CoronaVac	1369	13155	0.531 (0.498–0.567)	0.584 (0.547–0.625)	41.6 (37.5–45.3)
*2 doses only*					
All BNT162b2	109	6121	0.076 (0.063–0.093)	0.093 (0.077–0.114)	90.7 (88.6–92.3)
All CoronaVac	669	15140	0.210 (0.193–0.228)	0.252 (0.231–0.275)	74.8 (72.5–76.9)
*3 doses*					
All BNT162b2	12	2700	0.016 (0.009–0.029)	0.020 (0.011–0.035)	98.0 (96.5–98.9)
All CoronaVac	34	3794	0.035 (0.025–0.049)	0.045 (0.032–0.063)	95.5 (93.7–96.8)
B-B-C	0	30	–	–	–
C-C-B	6	962	0.023 (0.010–0.050)	0.028 (0.013–0.063)	97.2 (93.7–98.7)
***Aged 51–64 years***					
Unvaccinated	224	621	(Ref)	(Ref)	(Ref)
*1 dose only*					
BNT162b2	13	170	0.192 (0.106–0.348)	0.201 (0.105–0.383)	79.9 (61.7–89.5)
CoronaVac	61	341	0.442 (0.321–0.609)	0.513 (0.361–0.730)	48.7 (27.0–63.9)
*2 doses only*					
All BNT162b2	35	774	0.107 (0.073–0.157)	0.124 (0.082–0.186)	87.6 (81.4–91.8)
All CoronaVac	59	847	0.167 (0.121–0.229)	0.193 (0.137–0.272)	80.7 (72.8–86.3)
*3 doses*					
All BNT162b2	3	513	0.012 (0.004–0.039)	0.016 (0.005–0.050)	98.4 (95.0–99.5)
All CoronaVac	4	420	0.019 (0.007–0.052)	0.024 (0.009–0.067)	97.6 (93.3–99.1)
B-B-C	0	3	–	–	–
C-C-B	0	184	–	–	–
***Aged 18–50 years***					
Unvaccinated	49	108	(Ref)	(Ref)	(Ref)
*1 dose only*					
BNT162b2	2	33	0.145 (0.033–0.637)	0.101 (0.017–0.614)	89.9 (38.6–98.3)
CoronaVac	7	42	0.354 (0.145–0.868)	0.622 (0.226–1.708)	37.8 (−70.8–77.4)
*2 doses only*					
All BNT162b2	13	242	0.125 (0.063–0.249)	0.134 (0.062–0.290)	86.6 (71.0–93.8)
All CoronaVac	9	121	0.142 (0.062–0.327)	0.173 (0.069–0.435)	82.7 (56.5–93.1)
*3 doses*					
All BNT162b2	4	89	0.104 (0.036–0.304)	0.134 (0.043–0.421)	86.6 (57.9–95.7)
All CoronaVac	2	61	0.064 (0.014–0.285)	0.069 (0.013–0.362)	93.1 (63.8–98.7)
B-B-C	0	0	–	–	–
C-C-B	1	24	0.092 (0.012–0.723)	0.195 (0.023–1.670)	80.5 (−67.0–97.7)

OR: odds ratio; VE: vaccine effectiveness; CI: confidence interval; B-B-C: two doses of BNT162b2 followed by CoronaVac; C-C-B: two doses of CoronaVac followed by BNT162b2.

Adjusted for comorbidities (cancer, chronic kidney disease, respiratory disease, diabetes, cardiovascular disease, dementia), chronic medication use in past 90 days (renin-angiotensin-system agents, beta blockers, calcium channel blockers, diuretics, nitrates, lipid lowering agents, insulins, antidiabetic drugs, oral anticoagulants, antiplatelets, immunosuppressants).

**Table 3.** Vaccine effectiveness against COVID-19-related severe complications among individuals with different vaccination status by age group.

Vaccination status	Case	Control	Crude OR (95% CI)	Adjusted OR (95% CI)	VE % (95% CI)
***Aged 65 years and above***					
Unvaccinated	556	3291	(Ref)	(Ref)	(Ref)
*1 dose only*					
BNT162b2	38	357	0.554 (0.391–0.787)	0.609 (0.426–0.870)	39.1 (13.0–57.4)
CoronaVac	240	1740	0.747 (0.633–0.882)	0.841 (0.710–0.997)	15.9 (0.3–29.0)
*2 doses only*					
All BNT162b2	38	1189	0.148 (0.105–0.209)	0.179 (0.127–0.254)	82.1 (74.6–87.3)
All CoronaVac	167	2389	0.347 (0.288–0.418)	0.411 (0.339–0.497)	58.9 (50.3–66.1)
*3 doses*					
All BNT162b2	12	652	0.078 (0.043–0.140)	0.092 (0.051–0.166)	90.8 (83.4–94.9)
All CoronaVac	20	859	0.098 (0.062–0.156)	0.120 (0.075–0.192)	88.0 (80.8–92.5)
B-B-C	0	6	–	–	–
C-C-B	2	226	0.037 (0.009–0.151)	0.048 (0.012–0.195)	95.2 (80.5–98.8)
***Aged 51–64 years***					
Unvaccinated	71	227	(Ref)	(Ref)	(Ref)
*1 dose only*					
BNT162b2	11	60	0.498 (0.246–1.009)	0.652 (0.303–1.400)	34.8 (−40.0–69.7)
CoronaVac	29	128	0.688 (0.422–1.120)	0.923 (0.540–1.578)	7.7 (−57.8–46.0)
*2 doses only*					
All BNT162b2	18	405	0.128 (0.073–0.222)	0.170 (0.095–0.304)	83.0 (69.6–90.5)
All CoronaVac	38	403	0.274 (0.177–0.423)	0.329 (0.204–0.529)	67.1 (47.1–79.6)
*3 doses*					
All BNT162b2	4	233	0.047 (0.017–0.132)	0.057 (0.020–0.163)	94.3 (83.7–98.0)
All CoronaVac	7	201	0.094 (0.042–0.212)	0.124 (0.053–0.290)	87.6 (71.0–94.7)
B-B-C	0	3	–	–	–
C-C-B	2	78	0.070 (0.017–0.293)	0.114 (0.026–0.496)	88.6 (50.4–97.4)
***Aged 18–50 years***					
Unvaccinated	48	153	(Ref)	(Ref)	(Ref)
*1 dose only*					
BNT162b2	5	68	0.216 (0.081–0.576)	0.196 (0.065–0.593)	80.4 (40.7–93.5)
CoronaVac	11	49	0.680 (0.328–1.409)	0.661 (0.275–1.585)	33.9 (−58.5–72.5)
*2 doses only*					
All BNT162b2	27	404	0.217 (0.130–0.363)	0.217 (0.120–0.392)	78.3 (60.8–88.0)
All CoronaVac	11	160	0.200 (0.097–0.411)	0.222 (0.098–0.504)	77.8 (49.6–90.2)
*3 doses*					
All BNT162b2	7	111	0.196 (0.084–0.458)	0.245 (0.098–0.611)	75.5 (38.9–90.2)
All CoronaVac	2	54	0.099 (0.023–0.431)	0.122 (0.026–0.571)	87.8 (42.9–97.4)
B-B-C	0	1	–	–	–
C-C-B	1	20	0.166 (0.022–1.260)	0.261 (0.033–2.068)	73.9 (−106.8–96.7)

OR: odds ratio; VE: vaccine effectiveness; CI: confidence interval; B-B-C: two doses of BNT162b2 followed by CoronaVac; C-C-B: two doses of CoronaVac followed by BNT162b2.

Adjusted for comorbidities (cancer, chronic kidney disease, respiratory disease, diabetes, cardiovascular disease, dementia), chronic medication use in past 90 days (renin-angiotensin-system agents, beta blockers, calcium channel blockers, diuretics, nitrates, lipid lowering agents, insulins, antidiabetic drugs, oral anticoagulants, antiplatelets, immunosuppressants).

Vaccine effectiveness against COVID-19-related mortality after only one dose of BNT162b2 and CoronaVac were 73.5% (67.7–78.3) and 41.6% (37.5–45.3) respectively, in individuals aged ≥65. Greater risk reduction was observed after the second dose [BNT162b2: 90.7% (88.6–92.3), CoronaVac: 74.8% (72.5–76.9)], and further risk reduction was observed after the third dose [BNT162b2: 98.0% (96.5–98.9), CoronaVac: 95.5% (93.7–96.8)]. A similar dose–response relationship was observed in individuals aged 51–64 years. In individuals aged 18–50 years, vaccine effectiveness against COVID-19-related mortality remained consistently high after one to three doses of BNT162b2 [one-dose: 89.9% (38.6–98.3), two-doses: 86.6% (71.0–93.8), three-doses: 86.6% (57.9–95.7)], and increased with an increasing number of doses for CoronaVac [one-dose: 37.8% (−70.8–77.4), two-doses: 82.7% (56.5–93.1), three-doses: 93.1% (63.8–98.7)]. A similar relationship for vaccine effectiveness against severe complications was also observed (Table 3).

Vaccine effectiveness against COVID-19-related mortality and severe complications after one and two doses of BNT162b2 was higher than that of CoronaVac in individuals ≥ 65 years and 50–64 years. Nevertheless, vaccine effectiveness against COVID-19-related mortality and severe complications after receiving three doses of BNT162b2 or three doses of CoronaVac, were similarly high among all age groups. Due to a low number of fatal or severe complications after receiving two doses of BNT162b2 followed by CoronaVac, vaccine effectiveness for this combination could not be estimated in this study. Findings from the main analysis were robust to sensitivity analyses (Supplementary Tables 2–3).

## Discussion

This study is among the first to evaluate the real-world effectiveness of an mRNA (BNT162b2) and inactivated virus (CoronaVac) COVID-19 vaccination amidst an Omicron BA.2-dominant epidemic in a Chinese population. Our findings showed a significant risk reduction of COVID-19-related mortality and severe complications with one or more doses of either vaccine, demonstrating that both vaccines were effective in protecting against the Omicron BA.2 subvariant of SARS-CoV-2. In addition, our results suggested a clear dose–response relationship between the number of doses received and the protection against severe or fatal disease among individuals aged 51 years and above.

Evidence on the effectiveness of three doses of BNT162b2 against severe COVID disease caused by Omicron BA.2 is limited, especially in Asians. The reported vaccine effectiveness of two doses of BNT162b2 against hospitalization and death was 88.9% (95% CI: 52.1–97.4%) in Qatar when Delta was the dominant strain [33]. In terms of the Omicron strain, an earlier ecologic study in Hong Kong identified similarly high levels of protection against severe disease and mortality provided by two and three doses of COVID vaccines but was unable to account for potential confounding by underlying medical conditions or other factors [34]. A recent study in the United Kingdom specifically examined the effectiveness of BNT162b2 against the Omicron variant, but did not examine the effectiveness against severe disease caused by Omicron because only a very small number of patients who contracted Omicron were hospitalized in the UK [35]. Amongst the limited studies that recruited CoronaVac recipients, vaccine effectiveness against severe disease or COVID-related death in individuals who received two doses were reported to be 86.3% (95% CI: 84.5–87.9%) in Chile, [14] 81.3% (95% CI: 75.3–85.8%) in Brazil [15], and 100% (95% CI: 98.4–100.0%, reported as an overall value of four inactivated vaccines including CoronaVac) in China [16], however, these studies were conducted before the Omicron outbreak and none had investigated the effectiveness of the three-dose vaccine regimen against Omicron BA.2.

In this study, we report a consistently lower risk of complications and COVID-19-related mortality in recipients of BNT162b2 and CoronaVac, which is in line with prior studies that support the role of vaccines in reducing severe disease caused by Omicron [36,37]. When compared to the aforementioned studies that were conducted before the Omicron era, our study demonstrated considerably high vaccine effectiveness with three doses of either BNT162b2 or CoronaVac, suggesting that both vaccines are effective against the Omicron BA.2 variant.

Our findings of a positive dose–response relationship between the number of vaccine doses and risk reduction are largely aligned with observations from previous serological studies. In a previous study on SARS-CoV-2 vaccine immunogenicity in health-care workers in Hong Kong, antibody concentrations rose substantially after the first two doses of BNT162b2, but were low after the first dose and rose to moderate levels after the second dose of CoronaVac [17]. These findings were consistent with the apparently larger difference in vaccine effectiveness between the first dose of BNT162b2 and CoronaVac. From prior studies, serum samples obtained from recipients who completed three doses of BNT162b2 had significantly better neutralisation efficiency towards Omicron relative to samples from two-dose-only recipients [38]. Similarly, our findings showed a further risk reduction in severe complications and mortality in individuals with three doses of either vaccine compared to only receiving two doses, which reinforced the importance of a third dose in view of its effectiveness towards not only BA.1, but also BA.2 lineage of the Omicron variant.

There appears to be a slight difference between two-dose-BNT162b2 and two-dose-CoronaVac in preventing mortality, which is parallel to the results from an observational study conducted before the Omicron outbreak in Singapore, which reported higher effectiveness of two doses of BNT162b2 in preventing severe disease including oxygen supplementation in hospital, ICU admission and mortality, compared to that of CoronaVac [39]. However, such a difference in COVID-related complications and mortality was no longer detected between people who completed three doses of BNT162b2 and CoronaVac, during the Omicron BA.2 dominant period in the present study.

With respect to the mechanism of immune protection, it was postulated that adaptive immunity contributed by SARS-CoV-2-specific CD8+ T cells and CD4+ T cells is more important in predicting COVID disease severity [40]. According to a local serological study, T cell response was shown to be higher in two-dose-BNT162b2 recipients when compared to those who received two doses of CoronaVac [41]. Among those who received CoronaVac as the first two doses, more heterologous (BNT162b2) booster recipients developed positive T cell response than homologous booster recipients [42]. In contrast to these immunological findings, we did not demonstrate a significant difference between the risk of COVID-related mortality or complications, among those who completed three doses of CoronaVac versus BNT162b2. It was suggested that there were both quantitative and qualitative differences among the T-cell response induced by mRNA vaccine and inactivated vaccine [43]. Interestingly, a recent study reported that IFNγ-positive T cell response was higher in people who received two doses of BBIBP-CorV(Sinopharm) followed by BNT162b2, in comparison with people receiving three doses of BNT162b2, implying that inactivated vaccines might elicit a different, yet more diverse T cell response [44]. Whether T cell response or the level of neutralizing antibody can fully account for the effect against severe COVID remains to be elucidated, and more studies comparing mRNA and inactivated vaccines are warranted.

Vaccine hesitancy has been an issue, particularly among the older individuals in Hong Kong, contributed by various factors including attitude roots of negative perception of ageing, fatalistic risk attitudes and low health literacy [45]. With increasing awareness and the government’s efforts to facilitate vaccination amongst older individuals in Hong Kong and China [46,47], there has been a surge in the number of older people who received their very first dose of COVID-19 vaccine during the earlier phase of the current outbreak. Yet, the protection offered by such last-minute vaccination with only the first dose is unknown. In this study, a significant reduction in the risk of COVID-19-related mortality was already observed with one dose of either vaccine when compared to the unvaccinated population. Further risk reduction was noted after the second dose and the third dose. Notably, it was reported that vaccinated people had more robust antibody response than those who acquired a natural infection of Omicron [48]. Thus, it is crucial to offer vaccination to those who remain unvaccinated during the outbreak, which could reduce the risk of COVID-19 infection and mortality even if individuals are only able or willing to receive the first dose of COVID-19 vaccine.

To the best of our knowledge, this is among the first studies to provide real-world evidence on the important outcomes following mRNA (BNT162b2) and inactivated virus (CoronaVac) vaccinations during the Omicron BA.2 variant pandemic, where international data remains limited. Our findings reinforce the importance of a third dose to provide further risk reduction against the Omicron variant particularly for older adults. Nevertheless, this study has its limitations. First, similar to other retrospective epidemiological studies using electronic medical record data, the possible effects of residual confounding could not be ruled out. Second, the outcomes of this study were operationalized as mortality or severe complications within 28 or 14 days after diagnosis of infection respectively, where only temporal relationship was considered. We could not distinguish whether the patient died from complications due to COVID-19 or other causes since this would require a thorough causality assessment for each outcome event which was not feasible in research using vaccine registry and electronic medical record data with limited information in the automated database and a large number of events. Third, while some participants in the present study received a different type of vaccine as their third dose after the second dose, our sample size in this regard remains small and further studies are needed to elucidate the risks of COVID-19-related mortality and severe complications in this group.

## Conclusion

Both BNT162b2 and CoronaVac vaccination protected against COVID-19-related mortality and severe complications during an Omicron BA.2 epidemic, and the risk decreased with each additional vaccine dose. Therefore, it is imperative to promote vaccination with a third dose especially among vulnerable and older individuals.

## Supplementary Material

Supplemental Material
