# Supplementary material for: Effectiveness of BNT162b2 and CoronaVac vaccinations against mortality and severe complications after SARS-CoV-2 Omicron BA.2 infection: a case–control study
Source: Emerg Microbes Infect. 2022 Oct 9;11(1):2304–14. doi: 10.1080/22221751.2022.2114854 (PMC9553171; doi:10.1080/22221751.2022.2114854)
Supplement: Supplemental Material [file TEMI_A_2114854_SM2128.docx]

**Supplementary Table 1. Vaccination programme priority groups rollout schedule in Hong Kong**

| Order of expansion | Date of rollout | Vaccination group |
| --- | --- | --- |
| First [1] | 26 Feb 2021 | 1. Healthcare workers and staff involved in anti-epidemic work 2. Persons aged 60 or above (and a maximum of 2 carers accompanying elderly people aged above 70) 3. Residents and staff of residential care homes for the elderly and persons with disabilities 4. People providing essential public services 5. People providing cross-boundary transportation or working at control points and ports |
| Second [2] | 9 Mar 2021 | 1. Staff of food and beverage premises, markets, supermarkets, convenience stores, couriers and takeaway delivery (including takeaway food delivery) 2. Staff of local public transport service operators 3. Registered construction workers 4. Staff of property management 5. Teachers and school staff 6. Staff of the tourism industry 7. Staff of scheduled premises under the Prevention and Control of Disease (Requirements and Directions) (Business and Premises) Regulation |
| Third [3] | 16 Mar 2021 | 1. People aged between 30 and 59 2. Students studying outside Hong Kong (aged 16 or above) 3. Domestic helpers |
| Fourth [4] | 23 Apr 2021 | 1. People aged 16 to 29 (≥18 for person receiving CoronaVac) |
| Fifth [5] | 14 Jun 2021 | 1. People aged 12 to 15 for BNT162b2 |
| Sixth [6] | 11 Nov 2021 | 1. Eligible persons under certain groups can receive a third dose of COVID-19 vaccine free of charge |
| Seventh [7] | 23 Nov 2021 | 1. Members of the public who received two doses of the CoronaVac vaccine with the second dose received six months prior, irrespective of whether they belonged to certain groups, can reserve and receive a third dose of a COVID-19 vaccine |
| Eighth [8] | 2 Dec 2021 | 1. People aged 12 to 17 for CoronaVac |
| Ninth [9] | 1 Jan 2022 | 1. Provision of a third dose vaccination service to all eligible persons who have received two doses of the BNT162b2 vaccine with the second dose received six months prior |
| Tenth [10] | 21 Jan 2022 | 1. People aged 5 to 11 for CoronaVac |

**References:**

1. The Government of the Hong Kong Special Administrative Region. Government announces 2019 COVID-19 Vaccination Programme. Press Releases. 18 Feb 2021 (https://www.info.gov.hk/gia/general/202102/18/P2021021800767.htm?fontSize=1)

2. The Government of the Hong Kong Special Administrative Region. Government expands scope of priority groups and opens more CVCs. Press Releases. 8 Mar 2021 (https://www.info.gov.hk/gia/general/202103/08/P2021030800738.htm?fontSize=1)

3. The Government of the Hong Kong Special Administrative Region. Vaccination priority groups to be expanded to cover people aged 30 or above. Press Releases. 15 Mar 2021 (https://www.info.gov.hk/gia/general/202103/15/P2021031500626.htm?fontSize=1)

4. The Government of the Hong Kong Special Administrative Region. COVID-19 Vaccination Programme opens to persons aged 16 or above. Press Releases. 15 Apr 2021 (https://www.info.gov.hk/gia/general/202104/15/P2021041500565.htm?fontSize=1)

5. The Government of the Hong Kong Special Administrative Region. Secretary for Food and Health approves lowering age limit for receiving Comirnaty vaccine. 3 Jun 2021 (https://www.info.gov.hk/gia/general/202106/03/P2021060300652.htm?fontSize=1)

6. Third dose COVID-19 vaccination arrangements for persons under certain groups. Press Releases. 3 Nov 2021 (https://www.info.gov.hk/gia/general/202111/03/P2021110300536.htm)

7. SFH approves lowering age limit for receiving CoronaVac vaccine. Press Releases. 20 Nov 2021 (https://www.info.gov.hk/gia/general/202111/20/P2021112000292.htm)

8. Government extends third dose COVID-19 vaccination arrangements. Press Releases. 18 Nov 2021 (https://www.info.gov.hk/gia/general/202111/18/P2021111800310.htm)

9. Further expansion of COVID-19 vaccination arrangements from January 1. Press Releases. 24 Dec 2021 (https://www.info.gov.hk/gia/general/202112/24/P2021122400509.htm)

10. The Government of the Hong Kong Special Administrative Region. Arrangements for children aged 5 to 11 to receive COVID-19 vaccines. Press Releases. 20 Jan 2022 (https://www.info.gov.hk/gia/general/202201/20/P2022012000714.htm)

**Supplementary Table 2. Sensitivity analyses for COVID-19-related mortality**

***A) Limit vaccine exposure to at least 14 days since latest dose***

| **Vaccination status** | **Case** | **Control** | **Crude OR (95% CI)** | **Adjusted OR (95% CI)** | **VE % (95% CI)** |
| --- | --- | --- | --- | --- | --- |
| *Aged 65 years and above* | | | | | |
| Unvaccinated | 5452 | 33973 | (Ref) | (Ref) | (Ref) |
| *1 dose only* |  |  |  |  |  |
| BNT162b2 | 86 | 1564 | 0.292 (0.235-0.365) | 0.326 (0.261-0.409) | 67.4 (59.1-73.9) |
| CoronaVac | 910 | 10572 | 0.489 (0.454-0.528) | 0.546 (0.505-0.590) | 45.4 (41.0-49.5) |
| *2 doses only* |  |  |  |  |  |
| All BNT162b2 | 99 | 6338 | 0.077 (0.063-0.094) | 0.094 (0.076-0.115) | 90.6 (88.5-92.4) |
| All CoronaVac | 581 | 13455 | 0.231 (0.211-0.253) | 0.275 (0.251-0.301) | 72.5 (69.9-74.9) |
| *3 doses* |  |  |  |  |  |
| All BNT162b2 | 9 | 1794 | 0.021 (0.011-0.041) | 0.025 (0.013-0.049) | 97.5 (95.1-98.7) |
| All CoronaVac | 27 | 2676 | 0.045 (0.031-0.067) | 0.058 (0.039-0.085) | 94.2 (91.5-96.1) |
| B-B-C | 0 | 14 | - | - | - |
| C-C-B | 6 | 724 | 0.034 (0.015-0.077) | 0.042 (0.019-0.094) | 95.8 (90.6-98.1) |
| *Aged 51-64 years* | | | | | |
| Unvaccinated | 250 | 844 | (Ref) | (Ref) | (Ref) |
| *1 dose only* |  |  |  |  |  |
| BNT162b2 | 10 | 136 | 0.221 (0.114-0.430) | 0.286 (0.141-0.577) | 71.4 (42.3-85.9) |
| CoronaVac | 42 | 300 | 0.437 (0.305-0.627) | 0.500 (0.339-0.737) | 50.0 (26.3-66.1) |
| *2 doses only* |  |  |  |  |  |
| All BNT162b2 | 33 | 880 | 0.111 (0.076-0.163) | 0.131 (0.087-0.197) | 86.9 (80.3-91.3) |
| All CoronaVac | 59 | 916 | 0.186 (0.136-0.253) | 0.219 (0.157-0.306) | 78.1 (69.4-84.3) |
| *3 doses* |  |  |  |  |  |
| All BNT162b2 | 2 | 353 | 0.015 (0.004-0.062) | 0.019 (0.005-0.077) | 98.1 (92.3-99.5) |
| All CoronaVac | 3 | 311 | 0.023 (0.007-0.074) | 0.030 (0.009-0.097) | 97.0 (90.3-99.1) |
| B-B-C | 0 | 3 | - | - | - |
| C-C-B | 0 | 130 | - | - | - |
| *Aged 18-50 years* | | | | | |
| Unvaccinated | 52 | 137 | (Ref) | (Ref) | (Ref) |
| *1 dose only* |  |  |  |  |  |
| BNT162b2 | 0 | 26 | - | - | - |
| CoronaVac | 7 | 36 | 0.475 (0.197-1.146) | 0.755 (0.265-2.152) | 24.5 (-115.2-73.5) |
| *2 doses only* |  |  |  |  |  |
| All BNT162b2 | 14 | 258 | 0.151 (0.078-0.291) | 0.158 (0.076-0.327) | 84.2 (67.3-92.4) |
| All CoronaVac | 8 | 133 | 0.137 (0.057-0.328) | 0.163 (0.063-0.421) | 83.7 (57.9-93.7) |
| *3 doses* |  |  |  |  |  |
| All BNT162b2 | 3 | 66 | 0.132 (0.040-0.439) | 0.179 (0.050-0.645) | 82.1 (35.5-95.0) |
| All CoronaVac | 2 | 48 | 0.109 (0.025-0.474) | 0.105 (0.021-0.540) | 89.5 (46.0-97.9) |
| B-B-C | 0 | 0 | - | - | - |
| C-C-B | 1 | 16 | 0.147 (0.018-1.184) | 0.337 (0.036-3.188) | 66.3 (-218.8-96.4) |

***B) Test-negative case control among hospitalised patients***

| **Vaccination status** | **Case** | **Control** | **Crude OR (95% CI)** | **Adjusted OR (95% CI)** | **VE % (95% CI)** |
| --- | --- | --- | --- | --- | --- |
| *Aged 65 years and above* | | | | | |
| Unvaccinated | 3252 | 20643 | (Ref) | (Ref) | (Ref) |
| *1 dose only* |  |  |  |  |  |
| BNT162b2 | 78 | 1622 | 0.275 (0.218-0.347) | 0.287 (0.227-0.363) | 71.3 (63.7-77.3) |
| CoronaVac | 1045 | 8344 | 0.777 (0.720-0.838) | 0.798 (0.739-0.862) | 20.2 (13.8-26.1) |
| *2 doses only* |  |  |  |  |  |
| All BNT162b2 | 73 | 4241 | 0.096 (0.076-0.122) | 0.105 (0.083-0.133) | 89.5 (86.7-91.7) |
| All CoronaVac | 470 | 8423 | 0.323 (0.291-0.358) | 0.353 (0.319-0.392) | 64.7 (60.8-68.1) |
| *3 doses* |  |  |  |  |  |
| All BNT162b2 | 9 | 1460 | 0.031 (0.016-0.059) | 0.036 (0.018-0.069) | 96.4 (93.1-98.2) |
| All CoronaVac | 24 | 1908 | 0.064 (0.042-0.096) | 0.069 (0.046-0.103) | 93.1 (89.7-95.4) |
| B-B-C | 0 | 62 | - | - | - |
| C-C-B | 3 | 583 | 0.025 (0.008-0.079) | 0.029 (0.009-0.089) | 97.1 (91.1-99.1) |
| *Aged 51-64 years* | | | | | |
| Unvaccinated | 159 | 553 | (Ref) | (Ref) | (Ref) |
| *1 dose only* |  |  |  |  |  |
| BNT162b2 | 13 | 93 | 0.447 (0.242-0.825) | 0.504 (0.268-0.946) | 49.6 (5.4-73.2) |
| CoronaVac | 48 | 263 | 0.591 (0.409-0.852) | 0.651 (0.446-0.949) | 34.9 (5.1-55.4) |
| *2 doses only* |  |  |  |  |  |
| All BNT162b2 | 24 | 568 | 0.120 (0.075-0.192) | 0.138 (0.086-0.222) | 86.2 (77.8-91.4) |
| All CoronaVac | 38 | 534 | 0.208 (0.141-0.307) | 0.239 (0.160-0.357) | 76.1 (64.3-84.0) |
| *3 doses* |  |  |  |  |  |
| All BNT162b2 | 2 | 275 | 0.020 (0.005-0.084) | 0.026 (0.006-0.105) | 97.4 (89.5-99.4) |
| All CoronaVac | 3 | 244 | 0.033 (0.010-0.107) | 0.039 (0.012-0.126) | 96.1 (87.4-98.8) |
| B-B-C | 0 | 1 | - | - | - |
| C-C-B | 0 | 109 | - | - | - |
| *Aged 18-50 years* | | | | | |
| Unvaccinated | 34 | 91 | (Ref) | (Ref) | (Ref) |
| *1 dose only* |  |  |  |  |  |
| BNT162b2 | 0 | 24 | - | - | - |
| CoronaVac | 4 | 25 | 0.346 (0.107-1.118) | 0.566 (0.137-2.340) | 43.4 (-134.0-86.3) |
| *2 doses only* |  |  |  |  |  |
| All BNT162b2 | 9 | 143 | 0.181 (0.077-0.422) | 0.202 (0.080-0.507) | 79.8 (49.3-92.0) |
| All CoronaVac | 5 | 58 | 0.259 (0.091-0.735) | 0.272 (0.089-0.835) | 72.8 (16.5-91.1) |
| *3 doses* |  |  |  |  |  |
| All BNT162b2 | 3 | 55 | 0.195 (0.057-0.668) | 0.231 (0.063-0.844) | 76.9 (15.6-93.7) |
| All CoronaVac | 2 | 33 | 0.185 (0.041-0.835) | 0.174 (0.034-0.891) | 82.6 (10.9-96.6) |
| B-B-C | 0 | 0 | - | - | - |
| C-C-B | 1 | 8 | 0.499 (0.058-4.283) | 0.750 (0.084-6.717) | 25.0 (-571.7-91.6) |

***C) Adjusted for use of antibacterial and antiviral agents in the past 7 days***

| **Vaccination status** | **Case** | **Control** | **Crude OR (95% CI)** | **Adjusted OR (95% CI)** | **VE % (95% CI)** |
| --- | --- | --- | --- | --- | --- |
| *Aged 65 years and above* | | | | | |
| Unvaccinated | 4860 | 26990 | (Ref) | (Ref) | (Ref) |
| *1 dose only* |  |  |  |  |  |
| BNT162b2 | 111 | 2218 | 0.244 (0.201-0.296) | 0.290 (0.236-0.356) | 71.0 (64.4-76.4) |
| CoronaVac | 1369 | 13155 | 0.531 (0.498-0.567) | 0.624 (0.581-0.669) | 37.6 (33.1-41.9) |
| *2 doses only* |  |  |  |  |  |
| All BNT162b2 | 109 | 6121 | 0.076 (0.063-0.093) | 0.104 (0.085-0.127) | 89.6 (87.3-91.5) |
| All CoronaVac | 669 | 15140 | 0.210 (0.193-0.228) | 0.282 (0.257-0.308) | 71.8 (69.2-74.3) |
| *3 doses* |  |  |  |  |  |
| All BNT162b2 | 12 | 2700 | 0.016 (0.009-0.029) | 0.023 (0.013-0.042) | 97.7 (95.8-98.7) |
| All CoronaVac | 34 | 3794 | 0.035 (0.025-0.049) | 0.050 (0.035-0.071) | 95.0 (92.9-96.5) |
| B-B-C | 0 | 30 | - | - | - |
| C-C-B | 6 | 962 | 0.023 (0.010-0.050) | 0.032 (0.014-0.072) | 96.8 (92.8-98.6) |
| *Aged 51-64 years* | | | | | |
| Unvaccinated | 224 | 621 | (Ref) | (Ref) | (Ref) |
| *1 dose only* |  |  |  |  |  |
| BNT162b2 | 13 | 170 | 0.192 (0.106-0.348) | 0.177 (0.089-0.351) | 82.3 (64.9-91.1) |
| CoronaVac | 61 | 341 | 0.442 (0.321-0.609) | 0.487 (0.332-0.715) | 51.3 (28.5-66.8) |
| *2 doses only* |  |  |  |  |  |
| All BNT162b2 | 35 | 774 | 0.107 (0.073-0.157) | 0.121 (0.078-0.188) | 87.9 (81.2-92.2) |
| All CoronaVac | 59 | 847 | 0.167 (0.121-0.229) | 0.212 (0.147-0.305) | 78.8 (69.5-85.3) |
| *3 doses* |  |  |  |  |  |
| All BNT162b2 | 3 | 513 | 0.012 (0.004-0.039) | 0.015 (0.005-0.049) | 98.5 (95.1-99.5) |
| All CoronaVac | 4 | 420 | 0.019 (0.007-0.052) | 0.027 (0.009-0.076) | 97.3 (92.4-99.1) |
| B-B-C | 0 | 3 | - | - | - |
| C-C-B | 0 | 184 | - | - | - |
| *Aged 18-50 years* | | | | | |
| Unvaccinated | 49 | 108 | (Ref) | (Ref) | (Ref) |
| *1 dose only* |  |  |  |  |  |
| BNT162b2 | 2 | 33 | 0.145 (0.033-0.637) | 0.045 (0.002-0.830) | 95.5 (17.0-99.8) |
| CoronaVac | 7 | 42 | 0.354 (0.145-0.868) | 0.545 (0.154-1.923) | 45.5 (-92.3-84.6) |
| *2 doses only* |  |  |  |  |  |
| All BNT162b2 | 13 | 242 | 0.125 (0.063-0.249) | 0.158 (0.067-0.371) | 84.2 (62.9-93.3) |
| All CoronaVac | 9 | 121 | 0.142 (0.062-0.327) | 0.237 (0.087-0.645) | 76.3 (35.5-91.3) |
| *3 doses* |  |  |  |  |  |
| All BNT162b2 | 4 | 89 | 0.104 (0.036-0.304) | 0.070 (0.014-0.344) | 93.0 (65.6-98.6) |
| All CoronaVac | 2 | 61 | 0.064 (0.014-0.285) | 0.051 (0.008-0.344) | 94.9 (65.6-99.2) |
| B-B-C | 0 | 0 | - | - | - |
| C-C-B | 1 | 24 | 0.092 (0.012-0.723) | 0.251 (0.028-2.228) | 74.9 (-122.8-97.2) |

OR: odds ratio; VE: vaccine effectiveness; CI: confidence interval; B-B-C: two doses of BNT162b2 followed by CoronaVac; C-C-B: two doses of CoronaVac followed by BNT162b2.

**Supplementary Table 3. Sensitivity analyses for COVID-19-related severe complications**

***A) Limit vaccine exposure to at least 14 days since latest dose***

| **Vaccination status** | **Case** | **Control** | **Crude OR (95% CI)** | **Adjusted OR (95% CI)** | **VE % (95% CI)** |
| --- | --- | --- | --- | --- | --- |
| *Aged 65 years and above* | | | | | |
| Unvaccinated | 647 | 4192 | (Ref) | (Ref) | (Ref) |
| *1 dose only* |  |  |  |  |  |
| BNT162b2 | 28 | 265 | 0.589 (0.394-0.880) | 0.668 (0.444-1.006) | 33.2 (-0.6-55.6) |
| CoronaVac | 182 | 1467 | 0.741 (0.619-0.886) | 0.840 (0.700-1.010) | 16.0 (-1.0-30.0) |
| *2 doses only* |  |  |  |  |  |
| All BNT162b2 | 37 | 1285 | 0.152 (0.107-0.214) | 0.181 (0.128-0.256) | 81.9 (74.4-87.2) |
| All CoronaVac | 150 | 2260 | 0.365 (0.301-0.442) | 0.433 (0.356-0.526) | 56.7 (47.4-64.4) |
| *3 doses* |  |  |  |  |  |
| All BNT162b2 | 10 | 440 | 0.112 (0.059-0.211) | 0.133 (0.070-0.252) | 86.7 (74.8-93.0) |
| All CoronaVac | 17 | 621 | 0.135 (0.082-0.222) | 0.164 (0.099-0.270) | 83.6 (73.0-90.1) |
| B-B-C | 0 | 3 | - | - | - |
| C-C-B | 2 | 176 | 0.056 (0.014-0.227) | 0.072 (0.018-0.292) | 92.8 (70.8-98.2) |
| *Aged 51-64 years* | | | | | |
| Unvaccinated | 85 | 315 | (Ref) | (Ref) | (Ref) |
| *1 dose only* |  |  |  |  |  |
| BNT162b2 | 8 | 55 | 0.467 (0.213-1.027) | 0.585 (0.246-1.392) | 41.5 (-39.2-75.4) |
| CoronaVac | 23 | 114 | 0.697 (0.413-1.174) | 0.926 (0.525-1.632) | 7.4 (-63.2-47.5) |
| *2 doses only* |  |  |  |  |  |
| All BNT162b2 | 17 | 446 | 0.127 (0.073-0.221) | 0.155 (0.086-0.278) | 84.5 (72.2-91.4) |
| All CoronaVac | 36 | 434 | 0.280 (0.183-0.429) | 0.322 (0.203-0.513) | 67.8 (48.7-79.7) |
| *3 doses* |  |  |  |  |  |
| All BNT162b2 | 4 | 161 | 0.082 (0.030-0.230) | 0.095 (0.033-0.274) | 90.5 (72.6-96.7) |
| All CoronaVac | 6 | 148 | 0.131 (0.055-0.311) | 0.154 (0.063-0.380) | 84.6 (62.0-93.7) |
| B-B-C | 0 | 3 | - | - | - |
| C-C-B | 1 | 62 | 0.053 (0.007-0.394) | 0.083 (0.011-0.625) | 91.7 (37.5-98.9) |
| *Aged 18-50 years* | | | | | |
| Unvaccinated | 55 | 209 | (Ref) | (Ref) | (Ref) |
| *1 dose only* |  |  |  |  |  |
| BNT162b2 | 3 | 47 | 0.223 (0.065-0.765) | 0.257 (0.069-0.956) | 74.3 (4.4-93.1) |
| CoronaVac | 7 | 50 | 0.510 (0.214-1.211) | 0.470 (0.159-1.386) | 53.0 (-38.6-84.1) |
| *2 doses only* |  |  |  |  |  |
| All BNT162b2 | 29 | 429 | 0.263 (0.161-0.430) | 0.263 (0.151-0.458) | 73.7 (54.2-84.9) |
| All CoronaVac | 11 | 155 | 0.254 (0.125-0.516) | 0.274 (0.125-0.599) | 72.6 (40.1-87.5) |
| *3 doses* |  |  |  |  |  |
| All BNT162b2 | 4 | 72 | 0.204 (0.070-0.589) | 0.239 (0.076-0.752) | 76.1 (24.8-92.4) |
| All CoronaVac | 2 | 43 | 0.145 (0.033-0.638) | 0.190 (0.040-0.893) | 81.0 (10.7-96.0) |
| B-B-C | 0 | 1 | - | - | - |
| C-C-B | 1 | 14 | 0.268 (0.034-2.088) | 0.431 (0.052-3.560) | 56.9 (-256.0-94.8) |

***B) Test-negative case control among hospitalised patients***

| **Vaccination status** | **Case** | **Control** | **Crude OR (95% CI)** | **Adjusted OR (95% CI)** | **VE % (95% CI)** |
| --- | --- | --- | --- | --- | --- |
| *Aged 65 years and above* | | | | | |
| Unvaccinated | 483 | 3473 | (Ref) | (Ref) | (Ref) |
| *1 dose only* |  |  |  |  |  |
| BNT162b2 | 32 | 354 | 0.606 (0.416-0.884) | 0.667 (0.455-0.977) | 33.3 (2.3-54.5) |
| CoronaVac | 214 | 1420 | 1.065 (0.894-1.270) | 1.101 (0.921-1.315) | -10.1 (-31.5-7.9) |
| *2 doses only* |  |  |  |  |  |
| All BNT162b2 | 36 | 1076 | 0.212 (0.150-0.302) | 0.232 (0.163-0.330) | 76.8 (67.0-83.7) |
| All CoronaVac | 158 | 1747 | 0.590 (0.486-0.717) | 0.640 (0.525-0.780) | 36.0 (22.0-47.5) |
| *3 doses* |  |  |  |  |  |
| All BNT162b2 | 11 | 481 | 0.134 (0.073-0.247) | 0.147 (0.080-0.272) | 85.3 (72.8-92.0) |
| All CoronaVac | 18 | 565 | 0.187 (0.115-0.304) | 0.205 (0.126-0.335) | 79.5 (66.5-87.4) |
| B-B-C | 0 | 9 | - | - | - |
| C-C-B | 2 | 202 | 0.058 (0.014-0.237) | 0.063 (0.016-0.256) | 93.7 (74.4-98.4) |
| *Aged 51-64 years* | | | | | |
| Unvaccinated | 66 | 329 | (Ref) | (Ref) | (Ref) |
| *1 dose only* |  |  |  |  |  |
| BNT162b2 | 10 | 48 | 0.876 (0.415-1.851) | 1.089 (0.494-2.401) | -8.9 (-140.1-50.6) |
| CoronaVac | 26 | 106 | 1.117 (0.672-1.856) | 1.233 (0.720-2.111) | -23.3 (-111.1-28.0) |
| *2 doses only* |  |  |  |  |  |
| All BNT162b2 | 16 | 342 | 0.176 (0.096-0.322) | 0.201 (0.108-0.374) | 79.9 (62.6-89.2) |
| All CoronaVac | 36 | 335 | 0.486 (0.313-0.756) | 0.531 (0.333-0.846) | 46.9 (15.4-66.7) |
| *3 doses* |  |  |  |  |  |
| All BNT162b2 | 4 | 150 | 0.101 (0.035-0.289) | 0.115 (0.039-0.340) | 88.5 (66.0-96.1) |
| All CoronaVac | 6 | 170 | 0.153 (0.064-0.365) | 0.171 (0.070-0.417) | 82.9 (58.3-93.0) |
| B-B-C | 0 | 0 | - | - | - |
| C-C-B | 1 | 64 | 0.054 (0.007-0.409) | 0.066 (0.009-0.502) | 93.4 (49.8-99.1) |
| *Aged 18-50 years* | | | | | |
| Unvaccinated | 39 | 222 | (Ref) | (Ref) | (Ref) |
| *1 dose only* |  |  |  |  |  |
| BNT162b2 | 4 | 48 | 0.506 (0.171-1.501) | 0.565 (0.181-1.760) | 43.5 (-76.0-81.9) |
| CoronaVac | 10 | 29 | 1.729 (0.768-3.891) | 2.607 (1.062-6.403) | -160.7 (-540.3-6.2) |
| *2 doses only* |  |  |  |  |  |
| All BNT162b2 | 25 | 271 | 0.554 (0.323-0.950) | 0.513 (0.281-0.935) | 48.7 (6.5-71.9) |
| All CoronaVac | 10 | 120 | 0.453 (0.214-0.960) | 0.425 (0.182-0.993) | 57.5 (0.7-81.8) |
| *3 doses* |  |  |  |  |  |
| All BNT162b2 | 7 | 98 | 0.397 (0.168-0.941) | 0.381 (0.143-1.014) | 61.9 (-1.4-85.7) |
| All CoronaVac | 2 | 57 | 0.201 (0.046-0.882) | 0.227 (0.051-1.022) | 77.3 (-2.2-94.9) |
| B-B-C | 0 | 1 | - | - | - |
| C-C-B | 1 | 21 | 0.275 (0.036-2.132) | 0.290 (0.035-2.423) | 71.0 (-142.3-96.5) |

***C) Adjusted for use of antibacterial and antiviral agents in the past 7 days***

| **Vaccination status** | **Case** | **Control** | **Crude OR (95% CI)** | **Adjusted OR (95% CI)** | **VE % (95% CI)** |
| --- | --- | --- | --- | --- | --- |
| *Aged 65 years and above* | | | | | |
| Unvaccinated | 556 | 3291 | (Ref) | (Ref) | (Ref) |
| *1 dose only* |  |  |  |  |  |
| BNT162b2 | 38 | 357 | 0.554 (0.391-0.787) | 0.634 (0.441-0.910) | 36.6 (9.0-55.9) |
| CoronaVac | 240 | 1740 | 0.747 (0.633-0.882) | 0.857 (0.720-1.020) | 14.3 (-2.0-28.0) |
| *2 doses only* |  |  |  |  |  |
| All BNT162b2 | 38 | 1189 | 0.148 (0.105-0.209) | 0.186 (0.131-0.264) | 81.4 (73.6-86.9) |
| All CoronaVac | 167 | 2389 | 0.347 (0.288-0.418) | 0.427 (0.352-0.519) | 57.3 (48.1-64.8) |
| *3 doses* |  |  |  |  |  |
| All BNT162b2 | 12 | 652 | 0.078 (0.043-0.140) | 0.100 (0.055-0.180) | 90.0 (82.0-94.5) |
| All CoronaVac | 20 | 859 | 0.098 (0.062-0.156) | 0.126 (0.079-0.202) | 87.4 (79.8-92.1) |
| B-B-C | 0 | 6 | - | - | - |
| C-C-B | 2 | 226 | 0.037 (0.009-0.151) | 0.051 (0.013-0.208) | 94.9 (79.2-98.7) |
| *Aged 51-64 years* | | | | | |
| Unvaccinated | 71 | 227 | (Ref) | (Ref) | (Ref) |
| *1 dose only* |  |  |  |  |  |
| BNT162b2 | 11 | 60 | 0.498 (0.246-1.009) | 0.593 (0.265-1.330) | 40.7 (-33.0-73.5) |
| CoronaVac | 29 | 128 | 0.688 (0.422-1.120) | 0.946 (0.546-1.637) | 5.4 (-63.7-45.4) |
| *2 doses only* |  |  |  |  |  |
| All BNT162b2 | 18 | 405 | 0.128 (0.073-0.222) | 0.167 (0.091-0.306) | 83.3 (69.4-90.9) |
| All CoronaVac | 38 | 403 | 0.274 (0.177-0.423) | 0.330 (0.202-0.538) | 67.0 (46.2-79.8) |
| *3 doses* |  |  |  |  |  |
| All BNT162b2 | 4 | 233 | 0.047 (0.017-0.132) | 0.058 (0.020-0.169) | 94.2 (83.1-98.0) |
| All CoronaVac | 7 | 201 | 0.094 (0.042-0.212) | 0.138 (0.058-0.324) | 86.2 (67.6-94.2) |
| B-B-C | 0 | 3 | - | - | - |
| C-C-B | 2 | 78 | 0.070 (0.017-0.293) | 0.120 (0.028-0.524) | 88.0 (47.6-97.2) |
| *Aged 18-50 years* | | | | | |
| Unvaccinated | 48 | 153 | (Ref) | (Ref) | (Ref) |
| *1 dose only* |  |  |  |  |  |
| BNT162b2 | 5 | 68 | 0.216 (0.081-0.576) | 0.196 (0.063-0.606) | 80.4 (39.4-93.7) |
| CoronaVac | 11 | 49 | 0.680 (0.328-1.409) | 0.706 (0.293-1.700) | 29.4 (-70.0-70.7) |
| *2 doses only* |  |  |  |  |  |
| All BNT162b2 | 27 | 404 | 0.217 (0.130-0.363) | 0.222 (0.122-0.402) | 77.8 (59.8-87.8) |
| All CoronaVac | 11 | 160 | 0.200 (0.097-0.411) | 0.224 (0.097-0.519) | 77.6 (48.1-90.3) |
| *3 doses* |  |  |  |  |  |
| All BNT162b2 | 7 | 111 | 0.196 (0.084-0.458) | 0.240 (0.095-0.609) | 76.0 (39.1-90.5) |
| All CoronaVac | 2 | 54 | 0.099 (0.023-0.431) | 0.114 (0.024-0.554) | 88.6 (44.6-97.6) |
| B-B-C | 0 | 1 | - | - | - |
| C-C-B | 1 | 20 | 0.166 (0.022-1.260) | 0.254 (0.032-2.022) | 74.6 (-102.2-96.8) |

OR: odds ratio; VE: vaccine effectiveness; CI: confidence interval; B-B-C: two doses of BNT162b2 followed by CoronaVac; C-C-B: two doses of CoronaVac followed by BNT162b2.

**Supplementary Figure 1. Sample size and power estimation**

Remarks:

The number of matched sets needed achieve 80% power to detect vaccine effectiveness of 50 to 95% against the outcomes studied is presented above. With 63% of Hong Kong population received at least two vaccine doses by 1 Jan 2022, the number of matched sets (1 case and 10 controls) ranging from 5 to 78 were required to achieve 80% power to detect the odds ratios from 0.05 to 0.5 (corresponding to vaccine effectiveness of 50% to 95%) at 0.05 significance level [1-3].

References:

1. Lachin, John M. Sample size evaluation for a multiply matched case-control study using the score test from a conditional logistic (discrete Cox PH) regression model. Stat Med, 2008;27:2509-2523.

2. Lachin, John M. Biostatistical Methods: The Assessment of Relative Risks, Second Edition. John Wiley & Sons, 2011.

3. Tang, Yongqiang. Comments on ‘Sample size evaluation for multiply matched case-control study using the score test from a conditional logistic (discrete Cox PH) regression model. Stat Med, 2009;28:175-177.
